# Associated clinical factors for serious infections in patients with systemic lupus erythematosus

**DOI:** 10.1038/s41598-019-46039-5

**Published:** 2019-07-04

**Authors:** Ju-Yang Jung, Dukyong Yoon, Young Choi, Hyoun-Ah Kim, Chang-Hee Suh

**Affiliations:** 1 Department of Rheumatology, Ajou University School of Medicine, 164 Worldcup-ro, Yeongtong-gu, Suwon 16499 Korea; 2 Department of Biomedical Informatics, Ajou University School of Medicine, 164 Worldcup-ro, Yeongtong-gu, Suwon 16499 Korea

**Keywords:** Infectious diseases, Systemic lupus erythematosus, Risk factors

## Abstract

Infection occurs frequently in patients with systemic lupus erythematosus (SLE), and has been a major cause of morbidity and mortality. However, no large-scale comprehensive studies have estimated the effect of clinical characteristics on serious infection in actual clinical practice yet. We investigated the influence of clinical characteristics on serious infections using electronic medical records data. We conducted a nested case-control study. Patients with SLE who developed serious infection which needs hospitalization or intravenous antibiotics (n = 120) were matched to controls (n = 240) who didn’t. Odds ratios (OR) and 95% confidence intervals (CIs) for infection associated with clinical features were obtained by conditional logistic regression analyses. The conditional logistic regression analysis with adjustment showed that serositis (OR, 2.76; 95% CI, 1.33–5.74), hematologic involvement (OR, 2.53; 95% CI, 1.32–4.87), and use of higher than the low dose of glucocorticoids (GCs; >7.5 mg/d prednisolone-equivalent) (OR, 2.65; 95% CI, 1.31–5.34) were related to serious infections in SLE. Serositis, hematologic involvement, and use of higher than the low dose of GCs were associated with serious infections in patients with SLE.

## Introduction

Infection is a common and an important morbidity and a significant cause of death in patients with systemic lupus erythematosus (SLE)^1–4^. The defense immune function of these patients against bacteria, virus, and fungus has been found to be impaired, and opportunistic or serious infections develop frequently. Serious infections requiring hospitalization or intravenous antibiotics injection are associated with mortality and known to occur in 11–45% of patients with SLE^5^.

The defective phagocytosis of pathogens, unbalanced levels of pro- and anti-inflammatory cytokines, and lack of complements have been suggested as major causes of vulnerability of patients with SLE to infection^6–8^. Impaired immune functions cannot control the spread of pathogens and remove pathogens, effectively leading to continuation or worsening of infections. The manifestations and severity of SLE vary depending on the degree of uncontrolled autoimmune response or tissue invasion, which affects defense immunity. Most studies on infection in SLE have found correlations between disease activity markers or manifestations and infections, suggesting that imbalanced immune response with activated autoimmunity contributes to vulnerability of infection^9–11^.

Along with the intrinsic immune deregulations, the susceptibility to infection in terms of treatment also increases. The use of immunosuppressive drugs, including glucocorticoids (GCs), has improved the prognosis of SLE for several decades, making it possible to prevent patients with SLE from developing severe inflammation that either threatens their life or caused organ damage. Although immunosuppressive therapy is effective and essential in controlling disease activity, it has been found to be associated with cardiovascular disease, diabetes, osteoporosis and infection in patients with SLE^12,13^. The use of GCs has been shown to increase the risk of infection; however, some studies have not found a significant effect of GCs^1,14–19^. Hydroxychloroquine (HCQ) has been revealed to not only control disease activity and prevent flare up or serious manifestations such as nephritis but also to reduce serious infections in SLE^5,20^. Some studies have analyzed relationships between several immunosuppressive agents and infections in SLE, and concluded that the use of several immunosuppressive agents, such as mycophenolate mofetil, azathioprine, cyclophosphamide, showed no difference in the risk of infection and mortality^16^. Due to these conflicting evidences, the need of clinicians to know what is really happen in real world practice has been increasing.

However, no large-scale comprehensive studies have evaluated the effect of diverse clinical characteristics on serious infections in actual clinical practice yet. Therefore, we aimed to identify the clinical factors, including patterns in actual clinical practice, associated with serious infections in patients with SLE using the electronic medical records (EMRs) of a tertiary teaching hospital in Korea.

## Results

Among the total of 120 cases with infection, 93 (77.5%) were bacterial infections with 40 (25%) upper respiratory tract infections, 26 (21.7%) pneumonia, 24 (20%) sepsis, 22 (18.3%) urinary tract infections, and 16 (13.3%) gastrointestinal tract infections (Table 1). *Escherichia coli* and *Enterococcus faecium* were frequently found as the confirmed pathogens for the urinary tract infections. Twenty-four patients had sepsis caused by *E. coli, E. faecium, Pseudomonas sp., Acinetobacter, Streptococcus pneumoniae, methicillin-resistant Staphylococcus aureus, Micrococcus, Proteus*, and *Klebsiella*. In addition, seven and three patients had an infection caused by *Mycobacterium tuberculosis* and fungi, respectively. Nine patients expired during follow-up, and the cause of mortality in seven of them was infection.

**Table 1:** Origins of infection and pathogens.

Origin of Infection	N	Pathogens
Total cases	120	
Bacterial infection	93	
URI	30	Methicillin-susceptible *S. aureus* (1)
Pneumonia	26	*S. pneumoniae* (2), *Pseudomonas* sp. (2), *Acinetobact*er (4), Methicillin-resistant *S. aureus* (1)
Urinary tract infection	22	*E.coli* (7), *Enterococcus faecium* (8), Methicillin-resistant Coagulase negative *Staphylococcus* (2), *Micrococcus* (1)
GI tract infection	16	Vancomycin resistant *E.coli* (1)
Cellulitis	8	Methicillin-susceptible *S. aureus* (1), *Citrobacter and Serratia* (1)
PID	6	*E.coli* (1), *Enterococcus faecium* (1),
Septic arthritis	3	Methicillin-susceptible *S. aureus* (1)
Sepsis	24	*E.coli* (10), *Enterococcus faecium* (4), *Pseudomonas* sp.(2), *Acinetobacter* (9), *S. pneumoniae* (1), Methicillin-resistant *S. aureus* (5), *Proteus* (1), *Klebsiella* (1)
Mycobacterial infection	7	*Mycobacterium tuberculosis* (7)
Fungal infection	3	*Trichosporon asahii* (1), *Aspergillus* (1), *Candida* (1)
Viral infection	1	*Varicella zoster* (1)

URI: upper respiratory infection, GI: gastrointestinal, PID: pelvic inflammatory disease.

**Figure 1:**
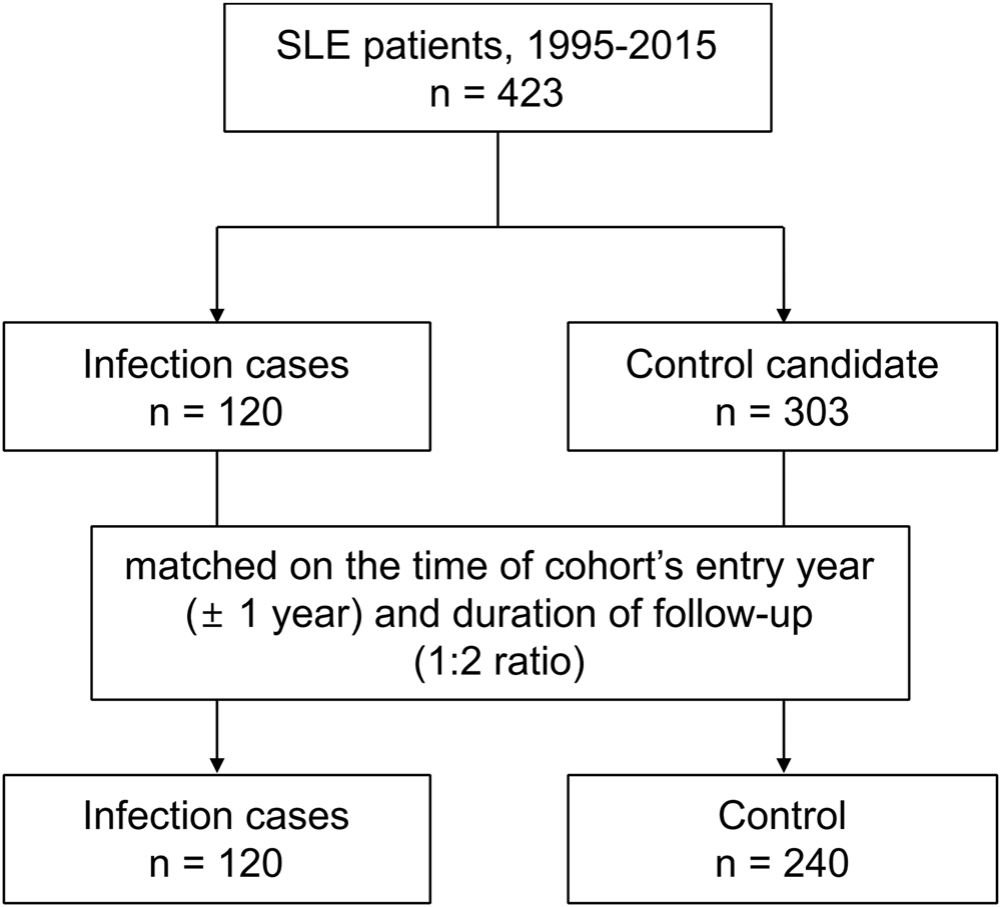
Dose-response analysis for the average daily dose of glucocorticoids (prednisolone-equivalent) in the patients with systemic lupus erythematosus and serious infection risks. The odds ratios were adjusted for the demographic factors, laboratory test results, comorbidity, and use of immunosuppressants. This figure shows that an increased risk of serious infections tended to be related to increased glucocorticoid doses. The patients who were not exposed to glucocorticoids were used as the reference group (gray dotted lines).

Table 2 displays the general characteristics of the infection and control groups. The total case-control sample included 360 patients with SLE admitted between 1995 and 2015. Each control (n = 240) was matched with two patients who developed infection (n = 120). The matching variables, including follow-up time and entry year, were distributed evenly between the groups. The average follow-up time did not differ between the infection (1,000 ± 1,043 d) and control groups (1,000 ± 1,041 d). Regarding the laboratory test results, the hemoglobin (11.1 ± 2.2/µL vs. 11.8 ± 1.7/µL, *p* = 0.001) and C3 (78.2 ± 40.2 mg/dL vs. 87.4 ± 39.5 mg/dL, *p* = 0.043) levels were lower in the infection group than in the control group. The patients with infection had higher incidences of clinical manifestations than the controls: nephritis (40.8% vs. 27.9%, *p* = 0.008), serositis (30.0% vs. 15.4%, *p* = 0.001), and hematologic involvement (41.7% vs. 22.9%, *p* < 0.001). Among the total patients, 314 (87.2%) patients took HCQ; those with infection less frequently took HCQ than the controls (80.0% vs. 90.8%, *p* = 0.003). The total and daily GC doses were not significantly different between the infection group (total 5,227.3 ± 6,190.7 mg; 10.4 ± 14.6 mg/d prednisolone-equivalent) and control group (total, 4,585.4 ± 6,875.7 mg; 7.2 ± 14.5 mg/d prednisolone-equivalent; *p* = 0.24 and *p* = 0.1, respectively). The number of patients who took higher than the low dose of GCs (>7.5 mg/d prednisolone-equivalent) (41.7%) was higher in the infection group than in the control group (26.3%, *p* = 0.001). There was no difference in the development of serious infection according to the immunosuppressive drugs (Table 3). As an ad-hoc analysis, we evaluated the dose response for the average daily dose of GCs and serious infection risk. We found that a higher GC dose tended to be associated with a higher risk of infections (Fig. 1).

**Table 2:** Characteristics of the study participants matched on follow-up time and year of entry.

Variables	Total (n = 360)	Infection cases (n = 120)	Controls (n = 240)
Age on diagnosis, years*	36.3 ± 13.0	36.0 ± 14.5	36.5 ± 12.1
Sex, female (%)*	323 (89.7)	106 (88.3)	217 (90.4)
WBC, /µL	6,708.1 ± 4,912.6	6,895.4 ± 5,038.8	6,614.5 ± 4,856.3
N/L ratio	7.6 ± 10.7	8.1 ± 12.7	7.4 ± 9.6
Hemoglobin, /µL	11.6 ± 1.9	11.1 ± 2.2	11.8 ± 1.7
Platelet, × 10^3^/µL	206.7 ± 103.5	212.3 ± 108.8	204.0 ± 100.9
ESR, mm/h	35.5 ± 25.8	37.2 ± 30.9	34.6 ± 22.9
Complement 3, mg/dL	84.3 ± 39.9	78.2 ± 40.2	87.4 ± 39.5
Complement 4, mg/dL	18.9 ± 11.7	18.4 ± 13.4	19.2 ± 10.8
Anti-dsDNA Ab, IU	28.0 ± 34.9	31.7 ± 35.8	26.1 ± 34.4
Oral ulcer, n (%)	296 (82.2)	102 (85.0)	194 (80.8)
Arthritis, n (%)	241 (66.9)	76 (63.6)	165 (68.8)
Nephritis, n (%)	244 (67.8)	49 (40.8)	67 (27.9)
Serositis, n (%)	73 (20.3)	36 (30.0)	37 (15.4)
Hematologic involvement, n (%)	105 (29.2)	50 (41.7)	55 (22.9)
Hydroxychloroquine, n (%)	314 (87.2)	96 (80.0)	218 (90.8)
Total dose of GCs, mg^a^	4,799.3 ± 6,653.5	5,227.3 ± 6,190.7	4,585.4 ± 6,875.7
Mean dose of GCs, mg/d^a^	8.3 ± 14.6	10.4 ± 14.6	7.2 ± 14.5
**Dose of GCs**			
≤7.5 mg/d^a^	247 (68.6)	70 (58.3)	177 (73.8)
>7.5 mg/d^a^	113 (31.4)	50 (41.7)	63 (26.3)
Immunosuppressive drugs	172 (47.8)	63 (52.5)	109 (45.4)
Survival time, days*	1,000 ± 1,040.2	1,000 ± 1,043.1	1,000 ± 1,040.9
Cohort entry, year*			
1995–1997	8 (2.2)	3 (2.5)	5 (2.1)
1998–2000	4 (1.1)	1 (0.8)	3 (1.3)
2001–2003	52 (14.4)	17 (14.2)	35 (14.6)
2004–2006	97 (26.9)	34 (28.3)	63 (26.3)
2007–2009	80 (22.2)	25 (20.8)	55 (22.9)
2010–2012	87 (24.2)	30 (25.0)	57 (23.8)
2013–2015	32 (8.9)	10 (8.3)	22 (9.2)

*Matching variable.

WBC: white blood cells, N/L ratio: Neutrophils/Lymphocyte ratio, ESR: erythrocyte sedimentation rate, dsDNA: double-strand deoxyribonucleic acid, Ab: antibody, GCs: glucocorticoids.

^a^Prednisolone-equivalent.

**Table 3:** Proportions of the patients taking the immunosuppressive drugs.

Drugs	Total	Infection case	Control
Azathioprine, n (%),	101 (28.1)	35 (29.2)	66 (27.5)
MMF, n (%)	37 (10.3)	12 (10.0)	25 (10.4)
Tacrolimus, n (%)	31 (8.6)	16 (13.3)	15 (6.3)
Methotrexate, n (%)	29 (8.1)	13 (10.8)	16 (6.7)
Cyclophosphamide, n (%)	27 (7.5)	12 (10.0)	15 (6.3)

MMF: mycophenolate mofetil.

Table 4 shows the results of the crude and adjusted conditional logistic regression analyses, which assessed the association between the clinical characteristics and infection. Based on the crude odd ratios (ORs), as the hemoglobin level increased by one unit, the risk of serious infection was shown to be 0.82 times lower (95% confidence interval (CI) 0.73–0.92). The patients with nephritis had a 1.97 times higher risk of serious infection (95% CI, 1.19–3.28), and those with serositis had a 2.61 times higher risk (95% CI, 1.47–4.61). Hematologic involvement increased, and the risk of serious infection was 2.48 times higher (95% CI, 1.52–4.06). The HCQ users had a 66% lower risk of serious infection (OR, 0.34; 95% CI, 0.16–0.69). The patients who took higher than the low dose of GCs had a 2.34 times higher risk of serious infection (95% CI, 1.38–3.95). The adjusted OR for the risk of serious infection in the patients with SLE who had serositis (OR, 2.76; 95% CI, 1.33–5.74) and hematologic involvement (OR, 2.53; 95% CI, 1.32–4.87) and in those who took higher than the low dose of GCs (OR, 2.65; 95% CI, 1.31–5.34) was significantly higher.

**Table 4:** The results of conditional logistic regression, assessing the association between serious infection and clinical characteristics in systemic lupus erythematosus.

Variables	COR	95% CI		*p*	AOR	95% CI		*p*
Age on diagnosis, years	1.00	0.98	1.02	0.73	1.00	0.98	1.02	0.898
**Sex**								
Male	1.00				1.00			
Female	0.81	0.4	1.62	0.548	0.9	0.38	2.14	0.793
WBC	1.00	1.00	1.00	0.604	1.00	1.00	1.00	0.234
N/L ratio	1.01	0.99	1.03	0.556	0.99	0.97	1.02	0.595
Hemoglobin, /µL	**0.82**	**0.73**	**0.92**	**0.001**	0.89	0.76	1.03	0.125
Platelet, x 10^3^/µL	1.00	1.00	1.00	0.462	1.00	1.00	1.01	0.183
ESR, mm/h	1.00	1.00	1.01	0.363	1.00	0.99	1.01	0.78
Complement 3, mg/dL	**0.99**	**0.99**	**1.00**	**0.043**	1.00	0.99	1.01	0.63
Complement 4, mg/dL	0.99	0.97	1.01	0.538	1.01	0.98	1.05	0.544
Anti-dsDNA Ab	1.00	1.00	1.01	0.162	1.00	0.99	1.01	0.833
**Oral ulcer, n (%)**								
No	1.00				1.00			
Yes	0.72	0.38	1.35	0.297	0.77	0.36	1.67	0.509
**Arthritis, n (%)**								
No	1.00				1.00			
Yes	1.28	0.8	2.05	0.297	1.38	0.78	2.47	0.273
**Nephritis, n (%)**								
No	1.00				1.00			
Yes	**1.97**	**1.19**	**3.28**	**0.008**	1.18	0.61	2.29	0.632
**Serositis, n (%)**								
No	1.00				1.00			
Yes	**2.61**	**1.47**	**4.61**	**0.001**	**2.76**	**1.33**	**5.74**	**0.007**
**Hematologic involvement, n (%)**								
No	1.00				1.00			
Yes	**2.48**	**1.52**	**4.06**	**<0.001**	**2.53**	**1.32**	**4.87**	**0.005**
**Hydroxychloroquine, n (%)**								
No	1.00				1.00			
Yes	**0.34**	**0.16**	**0.69**	**0.003**	0.42	0.17	1.03	0.06
**Mean GCs dose**								
≤7.5 mg/d^a^	1.00				1.00			
>7.5 mg/d^a^	**2.34**	**1.38**	**3.95**	**0.001**	**2.65**	**1.31**	**5.34**	**0.007**
**Immunosuppressive drugs, n (%)**								
No	1.00				1.00			
Yes	1.39	0.87	2.23	0.171	0.67	0.35	1.27	0.22

COR: crude odds ratio, CI: confidence intervals, AOR: adjusted odds ratio, WBC: white blood cells, N/L ratio: Neutrophils/Lymphocyte ratio, ESR: erythrocyte sedimentation rate, dsDNA: double-strand deoxyribonucleic acid, Ab: antibody, GCs: glucocorticoids.

^a^Prednisolone-equivalent.

## Discussion

In this nested case-control study, we analyzed 120 cases with infection and 240 controls who were divided by the occurrence of serious infection with matching variables, including age, sex, the follow-up time, and year of cohort entry. Our study found that serositis, hematologic involvement, and use of higher than the low dose of GCs (>7.5 mg/d prednisone-equivalent) were significantly associated with serious infection in patients with SLE.

Many studies showed the disease-related markers were associated with infection in SLE. A higher SLE disease activity index (SLEDAI), lower levels of complements, positive anti-dsDNA antibodies findings, nephritis at the time of SLE diagnosis, frequent flare-ups, or longer follow-up duration were significantly associated with infection in SLE^1,21–24^. Unlike previous studies, this study demonstrated no significant correlation with SLEDAI or anti-dsDNA antibody positivity, which represents disease activity. One of the reasons for the discrepancies with the findings of previous studies might be the difference in the year of data. Most previous studies have collected data in the 1990s; the treatment patterns and characteristics of disease in this period were different from those in the 2000s. In recent reports, infection had not been correlated with disease activity markers, but with treatment patterns^15,25^. In addition, this study collected serious infection cases requiring admission or intravenous antibiotics. Since the data on serious infections closely related to mortality were collected, the results might differ from those of studies that investigated general infections.

Hemolytic anemia is a hematologic involvement, along with leukopenia, lymphopenia, and thrombocytopenia^26,27^. Hematologic involvements are a predictor of an active disease or poor prognosis in SLE, and a study found the presence of hemolytic anemia increased the risk of mortality^28–30^. The neutrophil/lymphocyte ratio was reported as a good addictive marker for diagnosing infection in patients with SLE^31^. The white blood cell count, neutrophil/lymphocyte ratio, and platelet count were not significantly different between the infection and control groups; only the hemoglobin level was significantly different. A low hemoglobin level was found in the patients with SLE with hemolytic anemia, iron deficiency, and anemia of chronic disease. After adjustment, hematologic involvement was associated with serious infections in the patients with SLE. Vulnerability of the blood cells might be related to significant deficiency of protective immunity.

Pericardial effusion or pleural effusion are typical features in lupus flare-up, and manifestations which are assessed using chest X-ray or echocardiography when there is a possibility of diagnosis of SLE^32^. A study conducted on 5,414 patients showed 30% had serositis during their illness. The frequency of serositis had been reported to be 12–16% in European and Chinese patients with SLE^33^. An active disease status has been correlated with the development of serositis in SLE, and the risk factors for pleuritic and pericarditis were not different^34^. The association between serositis and infection has rarely been evaluated. Herein, serositis occurred more frequently in the patients with serious infections than in those without serious infections. Serositis could be an associated clinical factor for serious infections in SLE.

The use of >7.5 mg/d prednisolone-equivalent GCs was correlated with serious infections. A ≤7.5 mg/d prednisolone-equivalent dose is considered a low dose of GCs; thus, a higher than the low dose of GCs increases the risk of serious infections in SLE^13^. A higher dose of GCs is associated with risks of infection; a low dose GCs may provide a better risk-benefit for patients with SLE not only in the management of acute flare ups but also in the prevention of serious infections. This finding is consistent with that of other existing reports that studied diverse autoimmune inflammatory conditions, including rheumatoid arthritis, SLE, and inflammatory bowel disease^15,35,36^.

Findings on the impact of immunosuppressive drugs on the risk of infection in SLE has been conflicting. In some studies, immunosuppressants have been shown to increase the risk of infection; in others studies, they have not^1,16,17,22,24^. In general, the use of immunosuppressants was associated with infection when all types of infections were analyzed, but not when serious infections were investigated. Herein, serious infections were not associated with the use of immunosuppressants.

This study has some limitations. It lacked detailed information on infections or the tissue damages caused by SLE. However, the patients and controls were selected from the same EMR source; thus selection bias was minimized compared with a traditional case-based case-control study. In addition, all data, except for the symptoms, were extracted from the EMRs, which indicates that the probability of selection bias is low. The results of this study should be limited to cases of serious infection requiring hospitalization. Clinical manifestation in mild infection such as upper respiratory infection or cystitis, which are controlled by oral antibiotics, might be different. In addition, an admission to hospital could be another vulnerable factor against infection.

Our study findings suggest that serositis, hematologic involvement, and use of higher than the low dose GCs (>7.5 mg/d prednisolone-equivalent) are significantly correlated with serious infection in patients with SLE in actual clinical settings. Patients who have one or more of those clinical features require close monitoring. An appropriate dose of GCs is required to achieve favorable risk-benefit balance.

## Methods

### Data source and study participants

**Figure 2:**
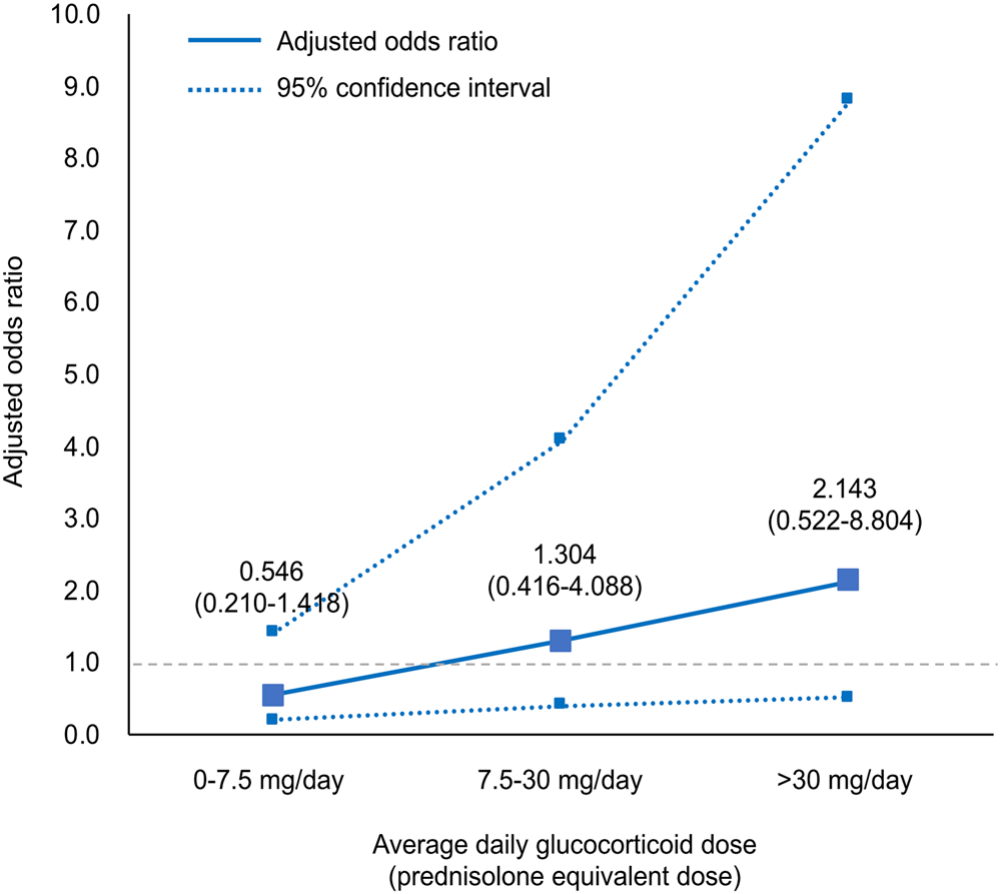
Study flow chart. Among patients with systemic lupus erythematosus in the study hospital, patients with serious infections needing hospitalization or intravenous antibiotic injection were matched with controls without serious infections.

EMR data from a tertiary teaching hospital in Korea (Ajou University Hospital) were used for the analysis (Fig. 2). The EMR data included information regarding unique de-identified numbers for the patients, age, sex, diagnostic codes, admission, discharge, laboratory test results, and prescribed medications/treatments. We selected patients with SLE admitted between 1995 and 2015. The patients who satisfied the Systemic Lupus International Collaborating Clinics/American College of Rheumatology classification criteria for SLE and received standard-of-care treatment for SLE were enrolled^37^. The results of the laboratory tests, including complete blood count, erythrocyte sedimentation rate (ESR), and complement levels (C3 and C4); antinuclear antibody test; and anti-double-stranded deoxyribonucleic acid (anti-dsDNA) antibody test were collected. Data on cumulative manifestations, including oral ulcer, malar rash, alopecia, arthritis, and renal disease, were also obtained. Comprehensive medication histories were obtained, including the use of GCs and immunosuppressant agents. The patients with SLE were observed from the date of first SLE diagnosis to the earliest incidence of infection, mortality, date of latest hospital visit, or end of the study period (May 31, 2015), whichever occurred first.

### Study design and definition of cases and controls

This study used a nested case-control design within a cohort to investigate the association between diverse clinical characteristics, including the use of GCs, and serious infection, which was defined as an infection needing intravenous antibiotic injections for more than 3 d and hospitalization. A nested case-control design was used because of the immortal time bias that can occur when measuring the cumulative dose of GCs. In this nested case-control study using the risk set sampling method, serious infection was identified using the following criteria: (1) the infection date was the prescribed date of antibiotics, which was considered the index date; (2) the period of antibiotic use was longer than 3 d; and (3) infections with antibiotic prescriptions within 7 d after the first antibiotic prescription were considered the same infection. Controls were selected from the cohort of patients with SLE who were at a risk of developing an infection at the time infection occurred in some patients; each control had to be alive at the time of infection occurrence in the cohort. The controls were randomly selected from the risk set of patients with SLE after they were matched for the time of entry year (±1 year) and duration of follow-up. For the 120 infection cases, they were individually matched at a 1:2 ratio (n = 240).

### Covariates

All covariates were assessed using all information before or at the index date. Demographic factors, laboratory test results, and comorbidity for infection were included in this study. The demographic factors included sex and age. The laboratory test results included the white blood cell count, neutrophil/lymphocyte ratio, hemoglobin level, platelet count, ESR, C3 and C4 levels, and anti-dsDNA antibody level. Because the subject enrolled in the study were SLE patients who were regularly managed in the hospital and all the laboratory test results were included in the components required for lupus management, there was no missing value. Comorbidity was identified from the diagnoses before the index date (oral ulcer, arthritis, nephritis, serositis, and hematologic involvement based on the diagnostic code). Immunosuppressants (e.g., azathioprine, mycophenolate mofetil, tacrolimus, methotrexate, and cyclophosphamide) were considered potential confounders for infection in the patients with SLE.

All GCs prescribed before the index date were identified from the EMRs. The dose of the GCs was converted to the equivalent dose of prednisolone (5 mg = methylprednisolone 4 mg = deflazacort 6 mg = triamcinolone 4 mg = hydrocortisone 20 mg)^38,39^. We defined the mean GC dose as the average dose across the prescription period.

### Statistical analysis

Descriptive statistics were used to analyze the patient characteristics, laboratory test results, comorbidity, and medication use in the patients with infection and matched controls. The groups were matched according to the follow-up time and year of cohort entry; however, these measures did not differ significantly. Because matched dataset was used for analysis, conditional logistic regression analysis was conducted to estimate the ORs and 95% CIs to assess the association between the clinical characteristics and serious infection in the patients with SLE. A *p*-value of <0.05 was considered statistically significant. All statistical analyses were conducted using the R software.

### Ethics approval and consent to participate

This study was approved and informed consent was waived by the Institutional Review Board of Ajou University Hospital (AJURB-MED-MDB-17-126) because the anonymized data was analyzed retrospectively.
